# Hemagglutinin protein of Peste des Petits Ruminants virus (PPRV) activates the innate immune response via Toll-like receptor 2 signaling

**DOI:** 10.1080/21505594.2021.1882246

**Published:** 2021-02-12

**Authors:** José M. Rojas, Elena Pascual, Sean R. Wattegedera, Miguel Avia, César Santiago, Verónica Martín, Gary Entrican, Noemí Sevilla

**Affiliations:** a Centro de Investigación en Sanidad Animal (CISA-INIA), Instituto Nacional de Investigación Agraria y Alimentaria, Madrid, Spain; b Moredun Research Institute, Edinburgh, Scotland, UK; c Centro Nacional de Biotecnología-CSIC, Madrid, Spain; d College of Medicine and Veterinary Medicine, University of Edinburgh, Edinburgh, Scotland, UK

**Keywords:** TLR2, morbillivirus, hemagglutinin, interleukin-8, dendritic cells, monocyte, macrophage, ERK

## Abstract

The toll-like receptor (TLR) family comprises both cell-surface and intracellular receptors that recognize different types of pathogen-associated molecular patterns (PAMPs) leading to the production of pro-inflammatory cytokines and subsequent development of adaptive immunity. TLR2 is a cell-surface receptor initially thought to act as a bacterial sentinel but also shown to recognize a number of viral glycoproteins. In this study, we sought to characterize the role of TLR2 in the activation of the immune response by peste des petits ruminants virus (PPRV), a morbillivirus of the *Paramixoviridae* family that causes an acute, highly contagious disease in goats and sheep. Using human embryonic kidney (HEK) 293 cells stably expressing human (h)TLR2 but lacking any other TLR, we found that PPRV induces IL-8 production in a dose-dependent manner. That activation is only observed in cells expressing hTLR2 and is greatly reduced when the receptor is blocked by pretreatment with specific antibody. We identified hemagglutinin (H) as the viral protein responsible of TLR2 activation by performing the same assays with purified recombinant mammalian-expressed H protein. Exogenous addition of recombinant H protein to cell culture induces high levels of interleukin (IL)-8 only in TLR2-expressing cells. Moreover, H engagement on TLR2 in the monocytic cell line THP-1 activates extracellular-signal-regulated kinase (ERK) signaling. Stimulation of primary ovine dendritic cells with either inactivated PPRV or purified recombinant H protein results in transcription of pro-inflammatory cytokines and the secretion of the Th1-polarizing cytokine IL-12. The role of these host immune mechanisms in the control of PPR is discussed.

## Introduction

Peste des petits ruminants (PPR) is a highly contagious acute disease affecting small ruminants, mainly goats and sheep [1], causing great economic losses. The causative agent, peste des petits ruminants virus (PPRV), belongs to the genus Morbillivirus of the family *Paramixoviridae*, which includes rinderpest virus, canine distemper virus and measles virus (MV), among others. The genome is a non-segmented, negative-strand RNA, with a length of approximately 16 Kb, encoding six structural proteins and two non-structural proteins. Among the structural proteins, the fusion (F) and the hemagglutinin (H) are two glycoproteins situated in the viral envelope [2]. The H protein is known to interact with two cell receptors to facilitate virus entry, the signaling lymphocyte activation molecule (SLAM) and Nectin-4 [3], expressed in immune cells and epithelium, respectively. In the course of PPRV infection, an efficient immune response is generated that clears the virus and confers long-time protection. However, prior to developing immunity a general immunosuppression is also induced that allows for secondary infections to take hold, a process mainly responsible for the high mortality observed during PPRV infection [4]. This immunosuppression is partially due to PPRV tropism for lymphocytes, causing severe lymphopenia and impairment of T-cell function during the acute phase of disease [4,5]. Furthermore, PPRV infection reduces the type I interferon (IFN) response [6–8] and enhances anti-inflammatory interleukin (IL)-10 production, thus contributing to immunosuppression and facilitating virus spread [5].

The innate immune system senses microbial structures (pathogen-associated molecular patterns: PAMPs) through pattern recognition receptors (PRRs). One of the families of PRRs is the Toll-like receptors (TLRs), with at least 13 members known in the mammalian system [9]. All the TLRs are type I transmembrane proteins, composed of an amino-terminal responsible for PAMP recognition, a transmembrane domain and a cytoplasmic carboxy-terminal domain that activates downstream signal transduction [10]. Ligand binding to TLR leads in many cases to the activation of apoptosis, phagocytosis, complement, and pro-inflammatory mediators, essential for the activation of an immune response [11,12]. Tissue and cell pattern expression of TLRs are useful tools to determine susceptibility to a pathogen. Thus, high levels of TLR3 and TLR7 correlate with increased inflammatory cytokine and reduced immunomodulatory cytokine expression during PPRV infection in Indian goat breeds and in water buffaloes [13]. Several viral proteins are known to activate TLRs, such as the activation of TLR4 by the Ebola glycoprotein [14], the respiratory syncytial virus fusion protein [15] and the dengue virus NS1 protein [16] that drive an inflammatory response; or the NS3 protein of hepatitis C [17], the NSP4 protein of rotavirus [18] or the H protein of MV [19] that activate TLR2. In the case of MV, the wild-type strain but not the vaccine strain activates cells via TLR2 by the interaction with the H protein. This activation of TLR2 induces pro-inflammatory cytokines in monocytic cells and the expression of CD150, the cellular receptor for MV, contributing to immune activation but also to viral spread.

The interaction of PPRV with TLRs, and their contribution to immune response activation or immunosuppression, is completely unknown. In this study, using human embryonic kidney (HEK) 293 cells expressing TLR2 but lacking other TLRs, we show that PPRV induces IL-8 by interacting with TLR2 in a dose-dependent manner. Moreover, purified PPRV H protein also activates IL-8 secretion, indicating that H protein interacts with TLR2. Monocytic THP1 cells are also activated by PPRV H protein, and, more relevantly, ovine dendritic cells show induction of IL-8 after engagement of PPRV H with TLR2. Thus, our work identified PPRV H protein as a new agonist of TLR2 and a potential trigger of innate immunity to PPRV infection.

## Material and methods

### Cells and viruses

HEK293T human embryonic kidney cells (ATCC CRL-3216) were maintained in Dulbecco’s modified Eagle’s medium (DMEM) supplemented with 10% fetal bovine serum (FBS) at 37°C. HEK293-Null and HEK293-hTLR2 cells (*Invivogen*) were maintained in the same medium supplemented with Normocin (50 mg/ml) and Blasticidin (10 mg/ml), both from *Invivogen*, according to the manufacturer’s instructions. THP-1 cells (ATCC TIB-202) were cultured and differentiated to macrophages with 50 ng/ml phorbol 12-myristate 13-acetate (PMA; Sigma) for 72 h and allowed to rest before use for 24 h as described [20]. Vero Dog SLAM (VDS) cells were obtained from Dr. Parida (The Pirbright Institute) and maintained as described [21]. PPRV strain Ivory Coast’89 (ICV’89) were kindly provided by Dr. Batten (The Pirbright Institute). Virus stocks were grown in VDS and tittered by plaque assay as described [22,23].

### Virus inactivation

1 x 10^6^ plaque-forming units (PFU)/ml of virus were incubated with freshly prepared 3 mM binary ethyleneimine (BEI) (Merck) for 24 h at 37°C, and the reaction stopped with 0.02 M sodium thiosulfate (Merck).

### Cloning of head domain of H protein

The sequence of the head domain of PPRV ICV’89 strain H protein (residues 156–610) was amplified by PCR using specific primers from a previously reported construction obtained in our lab (pSIREN-H) as template [22]. The fragment was BamHI/XhoI digested and ligated into a pDisplay vector (*Life Technologies*). A C-terminal stop codon was introduced to avoid protein fusion to the PDGFR transmembrane domain. The resulting construction (pDisplay-H-ICV) contains an N-terminal fusion of the protein with the murine Igκ-chain leader sequence, which directs it to the secretory pathway, followed by an HA tag to allow purification. The construction was proof-sequenced to confirm its identity.

### Expression and purification of H protein

Pre-confluent (30–40%) monolayers of HEK293T cells were transfected with pDisplay-H-ICV using a standard calcium phosphate transfection protocol described elsewhere. Following transfection, cells were cultured at 37°C for up to 8 days and supernatants were collected every 48 h. Supernatants were clarified by ultracentrifugation for 30 min at 3,000 x g, filtered through 0.45 µm filter (*Millipore*) and subsequently loaded onto an anti-HA sepharose coupled column at 0.5 ml/min flow rate. Column was washed with 5 column volumes of 10 mM Tris-HCl pH 8.3, 100 mM NaCl buffer to remove unspecific bound proteins. Protein was eluted with 20 mM Glycine pH 3 in 7 ml fractions. Fractions were analyzed by SDS-PAGE and the fraction showing the highest concentration was further analyzed by size exclusion chromatography using a Superdex 200 10/300 GL column (GE Healthcare) in the same buffer.

### Detection of PPRV H protein by western blot

Samples separated on polyacrylamide gels were incubated in transfer buffer (48 mM Tris–HCl, 39 mM glycine, 0.0375% SDS, and 20% methanol) and then transferred to methanol-activated PVDF membranes in a wet electroblotter (*BioRad*) for 1 h at 140 V. Blots were blocked with blocking solution (PBS containing 5% nonfat dry milk) and incubated with a primary rabbit antibody α-HA tag diluted in blocking solution for 2 h. Membranes were subsequently washed and incubated with a horseradish peroxidase-conjugated goat anti-rabbit antibody for 1 h. Membranes were finally developed with ECL chemiluminescence reagent (GE Healthcare).

### Reported gene assay and IL-8 ELISA

HEK293-Null and HEK293-hTLR2 cells were stimulated with the controls Pam3CSK4 (Invivogen) and Pam2CSK4 (Invivogen), synthetic diacylated lipopeptides that induce signaling through TLR2/1 and TLR2/6, respectively. In parallel, the indicated amounts of PPRV H protein or PPRV were added for stimulation. At 24 h post-stimulation, cell supernatants were collected and assayed for IL-8 content by IL-8 human ELISA kit (ThermoFisher), following manufacturer’s instructions. For blocking TLR2, cells were preincubated before stimulation with polyclonal antibody to human TLR2 (PAb hTLR2, Invivogen) at 1 μg/ml for 1 h at 37°C.

### THP-1 cell activation and western blot analysis

Differentiated THP-1 cells were starved in PBS for 2–4 h and activated for 15–30 min with recombinant PPRV-H (5 µg/ml) or TLR2 agonist Pam2CSK4 (5 µg/ml). Cell lysates were obtained, separated on SDS-PAGE and transferred to 0.45 µm PVDF membranes as described in [24]. Membranes were probed with anti-ERK (137F5), anti-phospho-ERK (D13.14.4E) (both from Cell Signaling), anti-GAPDH (GAPDH-71.1) (Sigma) and incubated with the appropriate secondary antibodies conjugated with horseradish peroxidase (HRP) (Donkey anti-Rabbit IgG or Sheep anti-Mouse IgG both from GE healthcare). Western blots were revealed with Pierce ECL plus western blotting substrate (Thermo Scientific) and band intensity was quantified using ImageJ software (National Institute of Health).

### Peripheral blood mononuclear cells isolation and dendritic cell differentiation

Blood was obtained from healthy donor ewes housed at the Department of Animal Reproduction (INIA, Madrid, Spain) and peripheral blood mononuclear cells (PBMCs) purified by standard centrifugation techniques using Ficoll gradient separation as described in Rojas et al. [22]. CD14^+^ cells were positively selected from PBMCs using MACS anti-human CD14 MicroBeads (Milteneyi Biotec) to purities >98%. CD14^+^ cells were cultured in RPMI 1640 (Gibco) supplemented with 10% FBS, 2 mM L-glutamine (Sigma-Aldrich), 5 mM HEPES (Sigma-Aldrich), and 5 μM 2-mercaptoethanol (Sigma-Aldrich) supplemented with 20 ng/ml of recombinant ovine GM-CSF (KingFisher) and ovine IL-4 (KingFisher). Fresh medium containing the same amount of cytokine was added every 3 days up to day 6 (mature phenotype).

### Stimulation of DCs and flow cytometric analyses

Ovine monocyte-derived DCs were stimulated during 24 h with different amounts of either inactivated PPRV or recombinant PPRV H. As control, cells were treated with TLR2 agonists Pam3CSK4 or Pam2CSK4. Cell supernatants were collected and analyzed by IL-12 content by ovine IL-12 p70 ELISA kit (RayBiotech), following manufacturer’s instruction. DCs were pelleted by centrifugation and a fraction was kept for RNA isolation and the other fraction was resuspended in staining buffer (PBS containing 2% FCS and 0.02% NaN_3_) for flow cytometry acquisition. Expression of cell surface molecules was quantified using the following antibodies: anti-bovine CD80 (clone IL-A159), CD86 (clone IL-A190) and MHC-II polymorphic DR/DQ (clone 49.1), anti-CD14 (clone TÜK4) (all from Biorad), anti-CD11b (clone MM12A), anti-CD11c (clone BAQ153A) (both from Kingfisher Biotech). To discriminate between live and dead cells, Viobility™ Fixable Dye (Miltenyi) was used. Cells were then washed twice in staining buffer and fixed in PBS 1% FBS, 4% paraformaldehyde. For phagocytosis assays, freshly isolated CD14^+^ monocytes or differentiated DCs were cultured for 3 h in presence of Crimson fluorescent (625/645) FluoSpheres Carboxylate-Modified 1.0 µm Microspheres (ThermoFisher) were added to the cultures at a 20:1 microsphere: cell ratio. Cells were then harvested and analyzed by flow cytometry. Cells were acquired using a FACSCalibur flow cytometer (Becton, Dickinson). Data were analyzed with FlowJo software (Three Star Inc.).

### Real-time PCR analyses

Total cellular RNA was isolated from DCs by treatment with Trizol (Invitrogen) according to the manufacturer’s instructions. RNAs were treated with DNAase to remove genomic DNA. Reverse transcription was performed using the Power SYBR Green Cells-to-Ct kit (Invitrogen) following the manufacturer’s instructions. To evaluate the levels of transcription of the different genes, real-time PCR was performed with a LightCycler® 480 System Instrument (Roche) using SYBR Green PCR core Reagents (Applied Biosystems) and specific primers (Table 1). Each sample was measured in triplicate under the following conditions: 10 min at 95°C, followed by 45 amplification cycles (15s at 95°C and 1 min at 60°C) and a dissociation cycle (30s at 95°C, 1 min 60°C and 30s at 95°C). The expression of individual genes was normalized to relative expression of GAPDH and the expression levels were calculated using 2^−ΔCt^ method, where ΔCt is determined by subtracting the GAPDH value from the target Ct. Negative control with no template was included in all the experiments. A melting curve for each PCR was determined by reading fluorescence every degree between 60°C and 95°C to ensure only a single product has been amplified.

**Table 1.** Real-time PCR primers used in this study. Gene names, forward and reverse primer sequences are indicated

Gene name	Reverse	Forward
IL1-β	TCTCTGTCCTGGAGTTTGCAT	CGAACATGTCTTCCGTGATG
IL-6	GGAGACAGCGAGTGGACTGAA	CCTCCAGGAACCCAGCTATG
IL-8	TCATGGATCTTGCTTCTCAGC	TGGGCCACACTGTGAAAAT
IP-10	AGCTGTCAGTAGCAAGGCTG	GCTCATCACCCTGAGCTGGTT

### Statistical analyses

Statistical analyses were performed with Graphpad Prism 6 software. Statistical tests used for data analyses are stated in the figure legends. Levels of significance were as follows: *p < 0.05; **p < 0.01; and ***p < 0.001.

## Results

### PPRV activates TLR2 signaling

**Figure 1.**
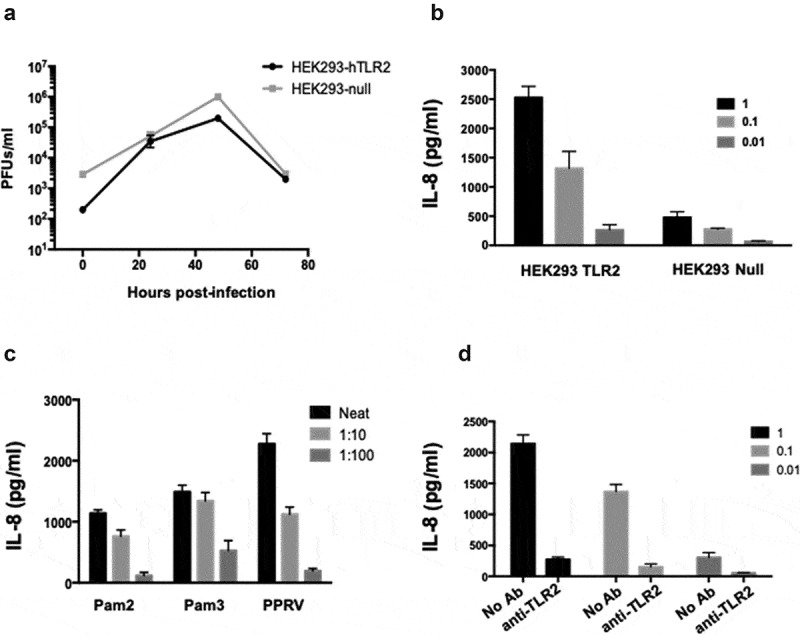
Activation of TLR2 by PPRV infection. HEK293 cells expressing hTLR2 were infected with PPRV ICV´89 at MOI of 1 PFU/cell. (a) Virus titers in supernatants of cells infected with PPRV ICV’89 were determined by plaque assay at different times post-infection. (b) Dose-dependent secretion of IL-8 from HEK293 cells expressing hTLR2 or null. Cells were infected with decreasing MOI of PPRV ICV’89 and the amount of cytokine secreted at 24 h pi were quantified by specific IL-8 ELISA. (c) Secretion of IL-8 in response to inactivated PPRV ICV’89. The amount of cytokine secreted in culture supernatant was compared in cells incubated with increased amounts of control agonists Pam_3_CSK_4_ (Pam3) or Pam_2_CSK_4_ (Pam2), or inactivated PPRV ICV’89 for 24 h. (d) Treatment with anti-hTLR2 antibody blocked IL-8 secretion. HEK293 cells expressing hTLR2 were treated with of anti-hTLR2 antibody (1 μg/ml) for 45 min prior to infection with PPRV ICV’89 at different MOIs. After 24 h of infection, cell-free medium was collected and IL-8 was quantified by IL-8 ELISA

To determine whether PPRV activates TLR2 signaling, we used HEK 293 cells stably expressing human TLR2 (hTLR2). Stimulation of TLR2 triggers a signaling cascade leading to the production of pro-inflammatory cytokines such as IL-8. First, we showed that PPRV ICV’89 was able to replicate in HEK293-hTLR2 and control HEK293-null cells (Figure 1a). Infection of HEK293-hTLR2 cells with PPRV ICV’89 at 1, 0.1, or 0.01 multiplicity of infection (MOI) induced release of IL-8 in a dose-dependent manner (Figure 1b). By contrast, control cells HEK293-null infected with PPRV ICV’89 did not significantly produce IL-8, indicating that PPRV activates the signaling cascade triggers after TLR2 engagement. Experiments in which HEK293-hTLR2 cells were treated with TLR2 agonists Pam3CSK_4_ (TLR2/1 ligand) or Pam2CSK_4_ (TLR2/6 ligand) confirmed that IL-8 production was mediated by TLR2 activation. Importantly, cell treated with inactivated PPRV ICV’89 also resulted in the induction of hTLR2-dependent signaling, indicating that PPRV replication is not required for signaling to occur (Figure 1c). To assure that PPRV induces IL-8 through hTLR2 activation in HEK293-hTLR2 cells, cells were treated with a neutralizing anti-HTLR2 antibody (or isotype control antibody) prior to infection with inactivated PPRV ICV’89 and secretion of IL-8 was evaluated by ELISA. Blocking of hTLR2 with anti-hTLR2 antibody prevented IL-8 production after PPRV infection (Figure 1d), confirming that IL-8 induction resulted from a surface interaction of hTLR2 with PPRV.

### PPRV H protein purification

**Figure 2.**
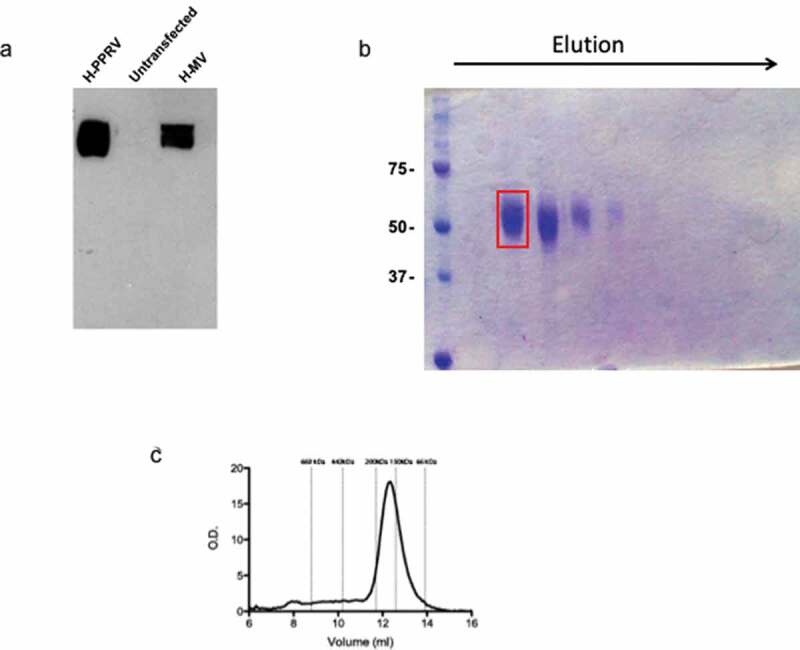
Expression and purification of PPRV H protein. (a) Analysis of the expression of H protein in the supernatant of transfected cells by Western blot using α-HA antibody (lane 1), together with an equivalent sample from untransfected cells (lane 2) or cells transfected with MV-H head domain plasmid (lane 3). (b) SDS-PAGE and Coomassie staining of the fractions eluted from the α-HA-Sepharose column. The red box highlights the presence of proteins with the predicted size in the fraction 2 of the eluate. (c) Size exclusion chromatogram of the previously obtained fraction 2 showing the presence of a single oligomeric species. Expected elution volumes of known protein calibration standards are indicated

Morbillivirus H protein interaction with their cellular receptor represents the first contact point between virus and cell [25,26]. Moreover, the H protein from MV activates cells via TLR2 [19]. Therefore, we studied the role of PPRV H protein in the activation of TLR2 signaling. To this end, we followed a similar strategy to that of Santiago *et al*. [27] to facilitate the production of a stable protein with a native conformation in which we specifically selected the residues comprising the predicted globular head domain of PPRV ICV’89 H protein based on sequence alignments with other paramyxoviruses. These residues (156–610) were cloned into a pDisplay vector to allow the secretion of the protein to the cell culture media and thus facilitate its folding and prevent aggregation. The expression of a protein of the expected size (~60 kDa) in cell supernatants was assessed by Western blotting using an α-HA antibody (Figure 2a). Recombinant H protein was subsequently purified using an α-HA antibody-coupled Sepharose column and the eluted fractions were analyzed by SDS-PAGE followed by Coomassie blue staining, showing a high protein concentration and purity (Figure 2b), however displaying rather diffuse bands which could account for different glycosylation states. Fraction 2 was selected for further experiments. To gain insight into the oligomeric state of the protein, this fraction was subjected to size exclusion chromatography using a Superdex 200 column. The chromatogram (Figure 2c) shows a single protein peak with a mobility of ~150 kDa, evidencing the presence of a single oligomeric state that most likely corresponds to a dimer with a slightly retarded mobility due to the previously mentioned modifications.

### PPRV H protein triggers TLR2 activation

**Figure 3.**
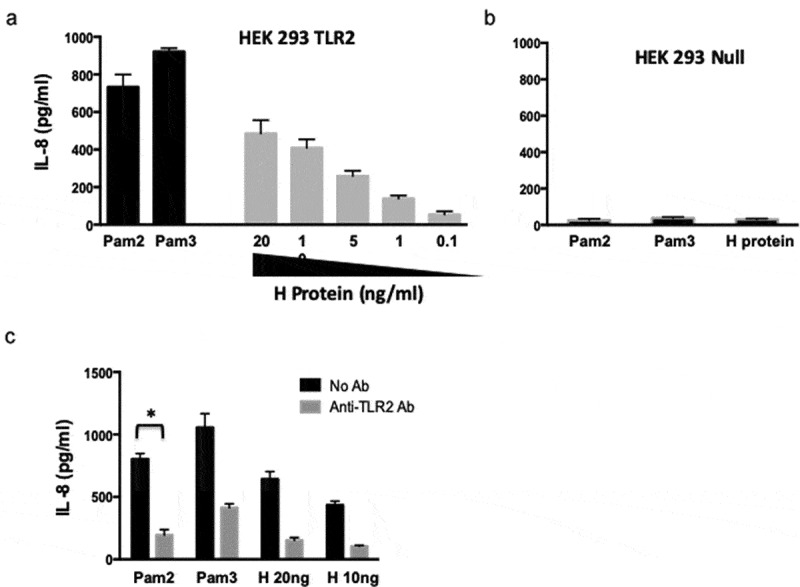
Activation of TLR2 by PPRV H protein. (a) HEK293 cells expressing hTLR2 were treated with increasing amounts of PPRV H protein or agonist Pam2 (5 μg/ml) or Pam3 (1 μg/ml) during 24 h. Supernatants were collected, and IL-8 concentration determined by ELISA. (b) HEK293-null cells were incubated with Pam2 (5 μg/ml) or Pam3 (1 μg/ml) or 20 ng/ml of PPRV H protein during 24 h. Cell-free medium was collected, and IL-8 concentration determined by ELISA. (c) HEK293 cells expressing hTLR2 were treated with anti-hTLR2 antibody (1 μg/ml) during 45 min prior to treatment with Pam2 or Pam3 or PPRV H protein. After 24 h, cell-free medium was collected and IL-8 quantified by ELISA. Black bars correspond to anti-hTLR2 antibody untreated cells and gray bars correspond to anti-hTLR2 antibody-treated cells. * p < 0.05, Student’s t-test

To determine the putative role of H protein in TLR2 activation, HEK293-hTLR2 cells were treated with different amounts of purified H protein (a range from 20–0.1 ng/ml). Treatment with PPRV H protein stimulated the TLR2 pathway in a dose-dependent manner as indicated by the secretion of IL-8 in culture supernatants (Figure 3a). Control HEK293-null cells did not show any IL-8 secretion in culture supernatants after PPRV H protein treatment (Figure 3b), suggesting that PPRV H protein activated the TLR2 pathway. To prove that PPRV H protein was activating the IL-8 secretion by the TLR2 pathway, HEK293-hTLR2 cells were incubated with anti-hTLR2 antibody prior to treatment with PPRV H protein or the controls Pam3CSK4 or Pam2CSK4 (Figure 3c). Anti-hTLR2 treatment reduced by 4-fold IL-8 secretion in HEK293-hTLR2 cells stimulated with 20 ng/ml of PPRV H protein (641 ± 49 versus 151 ± 19 pg/ml of IL-8), similarly to Pam2CSK4 (802 ± 37 versus 195 ± 35 pg/ml of IL-8) or Pam3CSK4 (1055 ± 90 versus 413 ± 25 pg/ml of IL-8). Thus, TLR2 blockade significantly reduced PPRV H protein-induced IL-8 secretion in HEK293-hTLR2 cells, indicating that the activation of this inflammatory pathway resulted from a surface interaction of PPRV H protein with TLR2.

### ERK signaling pathway is involved in PPRV H protein-TLR2 activation in monocyte/macrophages

**Figure 4.**
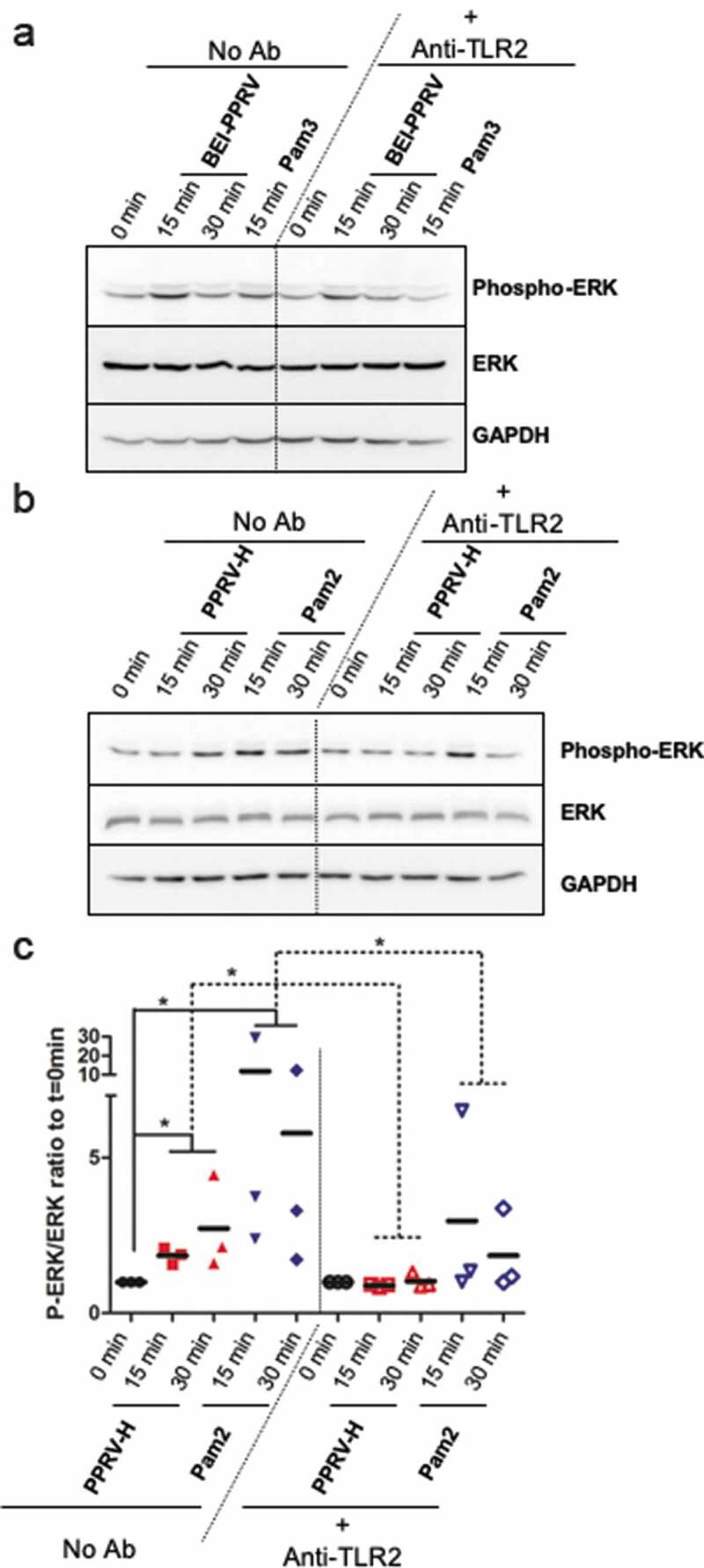
Inactivated PPRV- and H-protein-induced ERK phosphorylation is blocked by anti-TLR2 antibody treatment in macrophage-like differentiated THP-1 cells. (a) Representative western-blot of ERK phosphorylation in THP-1 cells differentiated into macrophages and serum starved for 2 h prior to stimulation with inactivated PPRV (BEI-PPRV) or the TLR2 agonist Pam3CSK4 (Pam3). Lysates were obtained prior to stimulation (0 min), and after 15 and 30 min stimulation with BEI-PPRV or 15 min after Pam3 stimulation. Neutralizing anti-TLR2 antibody was added 15 min prior to stimulation to assess TLR2 involvement. (b and c) Serum-starved differentiated THP-1 cells were treated with recombinant PPRV-H or the TLR2 agonist Pam2CSK4 (Pam2). Lysates were obtained prior to stimulation (0 min), and after 15- or 30-min stimulation with PPRV-H or Pam2. To confirm TLR2 involvement in ERK phosphorylation, cells were treated with neutralizing anti-TLR2 antibody 10 min prior to stimulation. (b) Representative western blot analysis of ERK phosphorylation following PPRV-H or positive control (Pam2) stimulation. (c) Phospho-ERK to total ERK (P-ERK/ERK) ratio for PPRV-H or Pam2 stimulation after 15 and 30 min in presence or absence of anti-TLR2 antibody was estimated by densitometry analysis in 3 independent experiments using ImageJ software. Data were normalized to baseline ERK phosphorylation ratio (i.e. prior to stimulation (t = 0 min)) and to loading control GAPDH. * p < 0.05; Two-way ANOVA with Sidak’s posttest (0 min vs PPRV-H/Pam2) and paired t-test (No Ab vs Anti-TLR2)

Monocyte/macrophages and dendritic cells are the main immune cells expressing TLR2 [28], and TLR activation triggers, among other cascades, the induction of the mitogen-activated protein kinase (MAPK) signaling pathways such as ERK. Therefore, we tested whether inactivated PPRV ICV’89 led to ERK phosphorylation in the human monocytic cell line THP-1 (Figure 4a). Macrophage-like differentiated THP-1 cells were stimulated for 15–30 min with inactivated PPRV ICV’89 (BEI-PPRV) or the TLR2 agonist Pam3CSK4 and ERK phosphorylation was assessed with phospho-specific antibodies by Western blot. Inactivated PPRV ICV’89 led to ERK phosphorylation in this macrophage model, and this as partially impaired by pretreatment with anti-TLR2 antibodies. This indicated that PPRV triggers ERK activation in immune cells expressing TLR2. To determine whether PPRV-H protein was involved in ERK activation via TLR2. THP-1 cells were stimulated for 15–30 min with 5 μg/ml of PPRV-H or the agonist Pam2CSK4. ERK phosphorylation was measured after PPRV-H stimulation by Western blot (Figure 4b). Densitometric analysis showed that PPRV-H treatment in macrophage-like differentiated THP-1 cells resulted in ERK phosphorylation (Figure 4c). To ensure that ERK phosphorylation is mediated by the interaction between H protein and TLR2, THP-1 cells were treated with neutralizing anti-TLR2 antibody prior to H treatment and ERK phosphorylation was assessed by Western blot (Figure 4). Treatment with anti-TLR2 antibody blocked ERK phosphorylation induced by H protein or Pam2CSK4 treatment, indicating that H engagement on TLR2 activates ERK signaling in macrophage-like differentiated THP-1 cells.

### PPRV H protein is involved in activating pro-inflammatory cytokines on ovine monocyte-derived dendritic cells

**Figure 5.**
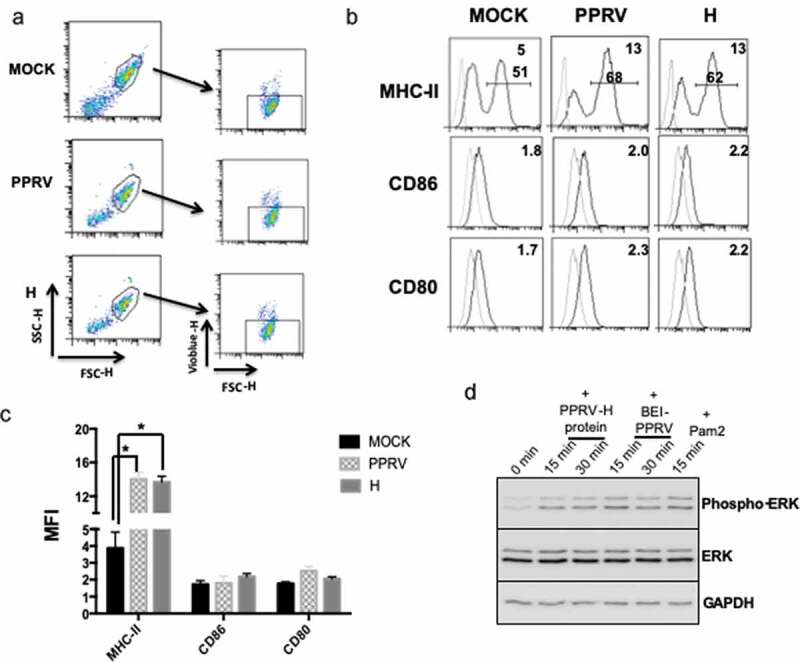
Inactivated PPRV and H protein treatment upregulate MHC-II on DCs and activates ERK phosphorylation. DCs were obtained from PBMCs following an established protocol (see Materials and Methods). (a) Representative dot plots showing the viability of inactivated PPRV- or H protein-treated DCs by staining with Vioblue-H. (b) Histograms showing the surface expression of MHC-II, CD86 or CD80 quantitated by flow cytometry in Mock-, inactivated PPRV- or H protein- treatment. Upper right corner numbers indicate the MFI and number on bars indicate percentage of MHC-II positive cells. Grey histograms are the isotype control. (c) Bars diagram indicating the MFI of MHC-II, CD80 and CD86 after Mock-, inactivated PPRV- or H protein-treatment. Results come from 4 different experiments. * Indicate statistically significant (p < 0.01). (d) Ovine DCs were treated with inactivated PPRV (BEI-PPRV), or recombinant PPRV-H protein, or the TLR2 agonist Pam2CSK4 (Pam2) for the indicated times. Lysates were obtained prior to stimulation (0 min), and after 15- or 30-min stimulation with BEI-PPRV, PPRV-H protein or Pam2 and ERK phosphorylation analyzed by Western blot. Representative Western blot data for Phospho-ERK, total ERK and GAPDH (used as loading control) are shown

Dendritic cells (DCs) are among the main cell population expressing TLR2 whose activation leads to pro-inflammatory cytokines release [29]. To determine whether inactivated PPRV or PPRV H protein was able to activate TLR2 on DCs in the natural host of the disease, ovine monocyte-derived DCs were generated by culture with GM-CSF and IL-4. Differentiated DCs showed increased expression of co-stimulatory molecules CD80 and CD86; of major histocompatibility complex (MHC)-II molecules; and of DC-related integrin CD11c when compared to freshly isolated CD14^+^ monocytes (Supplementary Figure 1A). Moreover, phagocytosis assays showed that differentiated DC displayed increased phagocytic activity when compared to monocytes (Supplementary Figure 1B). Ovine monocyte-derived DCs were thus treated with inactivated PPRV or recombinant PPRV H protein during 24 h to assess their activation. Inactivated PPRV was used to avoid viral replication that might affect DC function and viability. Neither inactivated PPRV nor PPRV H protein induced DC death (Figure 5a). We first evaluated by flow cytometry in live cells the expression of MHC-II and costimulatory molecules CD80 and CD86 (which are essential to DC function) to define the activation status of DCs following treatment with inactivated PPRV or H protein (Figure 5b). The data showed a significant increase in MHC-II expression after PPRV (14.02 ± 0.6 versus 3.9 ± 0.7 in MOCK cells) or H protein treatment (13.7 ± 0.5 versus 3.9 ± 0.7 in MOCK cells) (Figure 5b and c) but neither CD80 nor CD86 surface expression was up-regulated or down-regulated by the treatment. We also performed western-blot analysis of ERK phosphorylation in ovine DCs treated with PPRV-H protein or inactivated PPRV (Figure 5d). Both treatments resulted in ERK phosphorylation, which confirmed that PPRV-H protein and inactivated PPRV lead to ovine DC activation.

**Figure 6.**
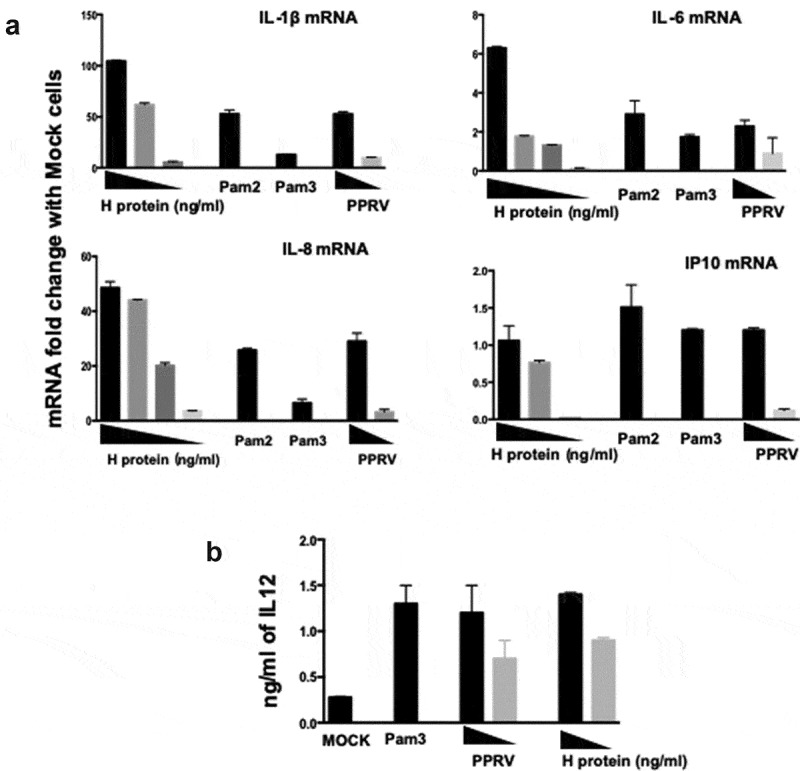
DCs produce pro-inflammatory cytokines and chemokines in response to H protein activation. Monocyte-derived DCs were stimulated with Pam3 (1 μg/ml), Pam 2 (5 μg/ml), increasing amounts of PPRV H protein (10, 5 and ng/ml) and increasing amounts of inactivated PPRV ICV’89 (1:10 and 1:100 dilutions). (a) At 24 h post-stimulation RNA was isolated, reverse transcribed, and real-time PCR carried out using specific primers (Table 1) for each cytokine or chemokine. Values are expressed as fold-change of mRNA values related to Mock cells. (b) At 24 h post-stimulation IL-12 secretion into the DC culture supernatants was determined by ELISA

One of the main outcomes of TLR activation is the production of cytokines. Therefore, we studied the transcription of pro-inflammatory cytokines IL-6 and IL-1β and chemokines IL-8 and interferon gamma-induced protein 10 (IP-10) through real-time PCR analyses after inactivated PPRV or H protein treatment of DCs. Data showed that pro-inflammatory cytokine transcription was highly activated in a dose-dependent manner after treatment with PPRV H protein, and at even higher levels than with inactivated PPRV (Figure 6a). Similar results were found with chemokines IL-8 and IP-10 (Figure 6a). Supernatants of treated monocyte-derived DCs were collected after 24 h of stimulation, and the amount of IL-12 released was determined by ELISA. DCs produced IL-12 after stimulation with synthetic Pam3CSK4, recombinant H protein, or inactivated PPRV (Figure 6b). Taken together, these data suggest that the interaction of PPRV H protein with TLR2 drives the transcription of pro-inflammatory cytokines in DCs from the natural host and provides a powerful signal for the generation of Th1 responses, as indicated by the IP-10 and IL-12 data.

## Discussion

The innate immune response that modulates adaptive immunity to clear PPRV infection and confer long-lasting immunity is mostly uncharacterized. Here, we describe for the first time that PPRV activates cellular signaling by TLR2 activation not only in established cell lines but also in sheep monocyte-derived DCs. Second, we show that purified PPRV H protein acts as an agonist of TLR2, activating the MAPK signaling pathway and inducing pro-inflammatory cytokine responses. Thus, this study provides evidence that activation of the TLR2 innate immune pathway is involved in the response to PPRV infection.

There is increasing evidence of TLR2 involvement in responses to viral infections (reviewed in [30]). We show that PPRV activates TLR2 in HEK293 cells expressing hTLR2, inducing release of IL-8. This secretion was significantly inhibited in the presence of antagonistic antibody to TLR2, supporting the evidence that PPRV recognition can be mediated by TLR2. Inactivated PPRV can also activate cells via TLR2, indicating that this activation is independent of newly synthesized viral gene products after infection. Activation of TLR2 by MV, a close related Morbillivirus, has been reported to be mediated by the H protein, stimulating the induction of pro-inflammatory cytokines in human monocyte DCs and the surface expression of CD150, the MV receptor [19]. Purified PPRV H protein activates TLR2 in a dose-dependent manner on HEK293T cells expressing hTLR2, inducing the secretion of IL-8. More interestingly, PPRV H protein also activates the TLR2-Myeloid differentiation primary response protein (MyD88) signaling pathway on the human monocytic cell line THP1 and increased proinflammatory cytokine transcript levels in ovine monocyte-derived DCs.

We have previously described [31] that PPRV infection *in vivo* produces antibodies that trigger antibody-dependent cell-mediated cytotoxicity (ADCC) of infected cells, and that the H protein is one of the main targets of this cytotoxic mechanism. Among innate immune cells, natural killer (NK) cells are one of the most important mediators of ADCC [32]. TLR2 signaling on NK cells has been shown to induce NK cell activation, a mechanism that can play a critical role in the control of viral infections such as vaccinia virus or herpes simplex virus [33,34]. The activation of TLR2 by PPRV H protein could therefore potentially lead to NK cell activation, which in turn could promote the activation of effector mechanisms on NK cells such as ADCC responses. NK cells, although well known for their potent antiviral and antitumoral activity, also function as important regulators of adaptive immunity during viral infections [35,36]. Activated NK cells can eliminate CD4^+^ T cells and CD8^+^ T cells in a perforin-mediated manner and thereby affect the T cell functions required for viral clearance [37,38]. Acute PPRV infection is characterized by an immunosuppression in which T cell is depleted and their activation is impaired [4,39]. The mechanism that drives PPRV immunosuppression and leads to viral spread and the development of opportunistic infections is completely unknown. Our data regarding PPRV-H-mediated TLR2 activation could thus hint at a new mechanism by which activated NK cells kill CD4^+^ and CD8^+^ T cells, contributing to PPRV immunosuppression. Further research is needed to demonstrate activation of NK cells by TLR2-H engagement and NK cell putative role in eliminating T cells.

Unlike TLR4, TLR2 is highly expressed on human monocytes, circulating blood DCs and monocyte-derived DCs [40]. In cattle, studies on the TLR expression repertoire of different DC subsets have shown that TLR2 was more highly expressed across all the DC subsets compared to other TLR genes [41]. Our results show that inactivated PPRV and PPRV H protein activates DCs, most likely through TLR2 engagement, resulting in the stimulation of pro-inflammatory cytokines/chemokines (IL-1β, IL-6 and IL-8) and the Th1-driving cytokines/chemokines IL-12 and IP-10. PPRV infection occurs through the respiratory tract, resulting in acute pneumonia [42,43], similar to other respiratory viruses. The inflammatory role of IL-6 and IL-1β in viral infections such as respiratory syncytial virus (RSV) and adenovirus has been reported to influence the susceptibility and severity of the respiratory disease [44]. TLR2 activation on monocytes and DCs triggers IL-6 trans-signaling, a mechanism in which IL-6 binds to soluble IL-6 R, forming a complex that is responsible for the pro-inflammatory properties of IL-6 [28,45]. Our data indicate that PPRV H protein activates DCs through TLR2 engagement to secrete pro-inflammatory cytokines, contributing to the excessive pulmonary inflammation resulting in tissue damage observed in PPRV infected animals. In addition, a mechanism of desensitization of TLR2 signaling pathway after secondary expose or re-stimulation by an agonist has been observed with bacterial components [46]. The interaction of PPRV H protein with TLR2 might induce tolerance to subsequent activation by bacterial components, resulting in high sensitivity to opportunistic infections associated with acute PPRV infection.

Activated DCs release cytokines that can direct the adaptive T cell response toward a Th1 or Th2 pattern [47]. Activation of DCs by TLR2 agonists stimulates IL-12 production from human monocyte-derived DCs [40], generating a Th1 response. Inactivated PPRV or PPRV H protein treated DCs stimulate IL-12 and IP-10, both involved in directing adaptive immunity to a Th1 response. Animals infected with PPRV develop a strong cellular and humoral response, both components required to control virus clearance and dissemination [4,48,49]. Our data suggest that activation of DCs through the PPRV H protein–TLR2 interaction initially primes a Th1 response, that could, at some point, switch to a Th2 response due to the role of IL-6 in maturating Th2 cells [50,51]. Therefore, initially, DCs activated by TLR2 will secrete IL-12, resulting in differentiation of naïve T cells into Th1 cells. At the same time, maturation of Th2 cells will start by the release of IL-6 by DCs, and these Th2 cells will produce IL-4 to generate more Th2 in an autocrine loop. These interconnections between Th1 and Th2 response will allow for a strong humoral as well as cellular response during PPRV infection.

Taken together, our data demonstrate a new recognition mechanism of PPRV by the innate immune system, through the interaction of PPRV-H protein with TLR2. This allows cells of the immune system to detect the infection and produce pro-inflammatory cytokines and Th-polarizing cytokines. The recognition of PPRV-H by TLR2 is not only important in understanding the onset of immunity to PPRV; it could also be relevant to the pathogenesis of PPRV. A deeper understanding of the host–pathogen interactions during PPRV infection will ultimately lead to improve treatment to this economically important viral disease.

## Supplementary Material

Supplemental Material
