# Supplementary figures and images for: Hemagglutinin protein of Peste des Petits Ruminants virus (PPRV) activates the innate immune response via Toll-like receptor 2 signaling
Source: Virulence. 2021 Feb 12;12(1):690–703. doi: 10.1080/21505594.2021.1882246 (PMC7889028; doi:10.1080/21505594.2021.1882246)

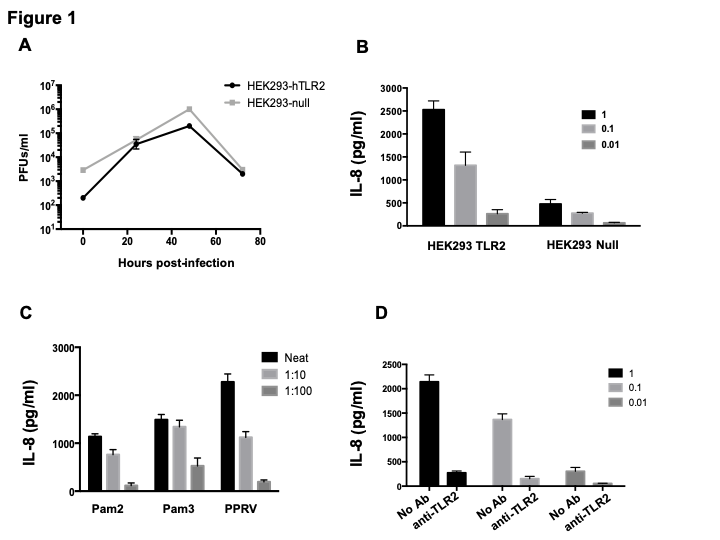

Supplement: Supplemental Material [file KVIR_A_1882246_SM4931.tiff]
